# Comparing the impact of atezolizumab plus bevacizumab and lenvatinib on the liver function in hepatocellular carcinoma patients: A mixed‐effects regression model approach

**DOI:** 10.1002/cam4.6726

**Published:** 2023-11-21

**Authors:** Takeshi Hatanaka, Satoru Kakizaki, Atsushi Hiraoka, Toshifumi Tada, Masashi Hirooka, Kazuya Kariyama, Joji Tani, Masanori Atsukawa, Koichi Takaguchi, Ei Itobayashi, Shinya Fukunishi, Kunihiko Tsuji, Toru Ishikawa, Kazuto Tajiri, Hironori Ochi, Satoshi Yasuda, Hidenori Toyoda, Chikara Ogawa, Keisuke Yokohama, Hiroki Nishikawa, Takashi Nishimura, Noritomo Shimada, Kazuhito Kawata, Hisashi Kosaka, Atsushi Naganuma, Yutaka Yata, Hideko Ohama, Hidekatsu Kuroda, Kazunari Tanaka, Takaaki Tanaka, Fujimasa Tada, Kazuhiro Nouso, Asahiro Morishita, Akemi Tsutsui, Takuya Nagano, Norio Itokawa, Tomomi Okubo, Taeang Arai, Michitaka Imai, Yohei Koizumi, Shinichiro Nakamura, Masaki Kaibori, Hiroko Iijima, Yoichi Hiasa, Masatoshi Kudo, Takashi Kumada, the Real‐life Practice Experts for HCC (RELPEC) Study Group, and HCC 48 Group (hepatocellular carcinoma experts from 48 clinics in Japan)

**Affiliations:** ^1^ Department of Gastroenterology Gunma Saiseikai Maebashi Hospital Maebashi Japan; ^2^ Department of Clinical Research National Hospital Organization Takasaki General Medical Center Takasaki Japan; ^3^ Department of Gastroenterology and Hepatology Gunma University Graduate School of Medicine Maebashi Japan; ^4^ Gastroenterology Center, Ehime Prefectural Central Hospital Matsuyama Japan; ^5^ Department of Internal Medicine, Japanese Red Cross Himeji Hospital Himeji Japan; ^6^ Department of Gastroenterology and Metabology Ehime University Graduate School of Medicine Matsuyama Japan; ^7^ Department of Gastroenterology, Okayama City Hospital Okayama Japan; ^8^ Department of Gastroenterology and Neurology Kagawa University Kita‐gun Japan; ^9^ Division of Gastroenterology and Hepatology, Department of Internal Medicine, Nippon Medical School Tokyo Japan; ^10^ Department of Hepatology, Kagawa Prefectural Central Hospital Takamatsu Japan; ^11^ Department of Gastroenterology, Asahi General Hospital Asahi Japan; ^12^ Department of Gastroenterology, Division of Hepatobiliary and Pancreatic Diseases Hyogo Medical University Nishinomiya Japan; ^13^ Center of Gastroenterology, Teine Keijinkai Hospital Sapporo Japan; ^14^ Department of Gastroenterology, Saiseikai Niigata Hospital Niigata Japan; ^15^ Department of Gastroenterology Toyama University Hospital Toyama Japan; ^16^ Center for Liver‐Biliary‐Pancreatic Disease, Matsuyama Red Cross Hospital Matsuyama Japan; ^17^ Department of Gastroenterology and Hepatology Ogaki Municipal Hospital Japan; ^18^ Department of Gastroenterology, Japanese Red Cross Takamatsu Hospital Takamatsu Japan; ^19^ Department of Gastroenterology Osaka Medical and Pharmaceutical University Osaka Japan; ^20^ Division of Gastroenterology and Hepatology, Otakanomori Hospital Kashiwa Japan; ^21^ Hepatology Division, Department of Internal Medicine II Hamamatsu University School of Medicine Hamamatsu Japan; ^22^ Department of Surgery Kansai Medical University Hirakata Japan; ^23^ Department of Gastroenterology, National Hospital Organization Takasaki General Medical Center Takasaki Japan; ^24^ Department of Gastroenterology, Hanwa Memorial Hospital Osaka Japan; ^25^ Department of Gastroenterology, Takarazuka City Hospital Takarazuka Japan; ^26^ Division of Gastroenterology and Hepatology, Department of Internal Medicine Iwate Medical University Iwate Japan; ^27^ Department of Gastroenterology and Hepatology Kindai University Faculty of Medicine Osaka Japan; ^28^ Department of Nursing Gifu Kyoritsu University Ogaki Japan

**Keywords:** ALBI score, atezolizumab plus bevacizumab, lenvatinib, liver function, mixed‐effects regression model

## Abstract

**Aim:**

This retrospective study compared the impact of atezolizumab plus bevacizumab (Atez/Bev) and lenvatinib (LEN) on the liver function in patients with hepatocellular carcinoma.

**Methods:**

We included 526 patients who received Atez/Bev and 731 who received LEN March 2018 and July 2022 in this study. We conducted a 1:1 propensity‐score‐matched analysis and identified 324 patients in each group for inclusion in the present analysis. Nonlinear mixed‐effects regression models were employed, allowing for the evaluation and inclusion of cases where treatment was interrupted due to disease progression, adverse events, or loss to follow‐up. These models were used to compare the ALBI score between the Atez/Bev and LEN groups.

**Results:**

Following propensity score matching, the mean ALBI scores in the Atez/Bev and LEN groups were −2.41 ± 0.40 and −2.44 ± 0.42 at baseline, and −2.17 ± 0.56 and −2.19 ± 0.58 at 12 weeks, respectively. Although the ALBI score significantly worsened during treatment in both groups (*p* < 0.001), there was no significant difference in the rate of ALBI score deterioration between the groups (*p* = 0.06). Subgroup analyses showed that LEN‐treated patients with BCLC advanced stage (*p* = 0.02) and those who initially received the full dose (*p* < 0.001) had a significantly greater worsening of ALBI score compared to Atez/Bev.

**Conclusions:**

Using a nonlinear mixed‐effects regression approach, which allowed for the inclusion of cases with treatment interruption, we found no significant difference in the trend of liver function deterioration between the Atez/Bev and LEN groups. Caution should be exercised for LEN‐treated patients with BCLC advanced stage or those receiving the full dose of LEN.

## 1 INTRODUCTION

Remarkable advances have been made in systemic therapy for advanced hepatocellular carcinoma (HCC). Currently, there are seven available regimens, including atezolizumab plus bevacizumab (Atez/Bev),^1^ tremelimumab plus durvalumab (Dur/Tre),^2^ and lenvatinib (LEN).^3^

The Imbrave150 trial^1^ compared the efficacy and safety of Atez/Bev to that of sorafenib and demonstrated that Atez/Bev was superior to sorafenib in terms of the progression‐free survival (PFS) and overall survival (OS). The HIMALAYA trial^2^ was designed to compare the Dur/Tre to sorafenib, and resulted in a significant improvement in the OS. Although there are no randomized control trials comparing efficacy and safety of Atez/Bev and Dur/Tre with those of LEN, Atez/Bev^4^, ^5^, ^6^ or Dur/Tre^4^ is recommended as the first‐line systemic treatments for advanced HCC. LEN is recommended for the first‐line treatment in accordance with Barcelona Clinical Liver Cancer (BCLC)^4^ and American Society of Clinical Oncology guidelines^5^ when there are contraindications to immunotherapies in advanced HCC patients, while it is recommended as the second‐line treatment after disease progression on Atez/Bev according to Japan Society of Hepatology guidelines.^6^ A network meta‐analysis showed that Atez/Bev was superior to LEN in terms of the OS with a hazard ratio (HR) of 0.63 (95% confidence interval [CI] 0.44–0.89), but not in terms of the PFS with a HR of 0.89 (95% CI 0.67–1.19).^7^

While there are many effective regimens available today, it is crucial to conduct sequential therapy to prolong the OS of advanced HCC patients. In particular, the liver function has a major influence on the OS.^8^ Although our colleagues previously reported the changes in ALBI score of patients during Atez/Bev^9^ and LEN,^10^ few studies have compared the effects of systemic agents on the liver function. Furthermore, it should be noted that previous studies on liver function have not considered cases of treatment interruption due to progression disease, adverse events (AEs) or loss to follow‐up, and excluding these cases could introduce significant statistical biases. To address this issue, we employed a nonlinear mixed‐effects regression model, which can account for these situations. Therefore, this real‐world study focused on comparing the rate of liver changes in patients who received Atez/Bev and LEN using nonlinear mixed‐effects regression model.

## 2 METHODS

### 2.1 Patients

**FIGURE 1:**
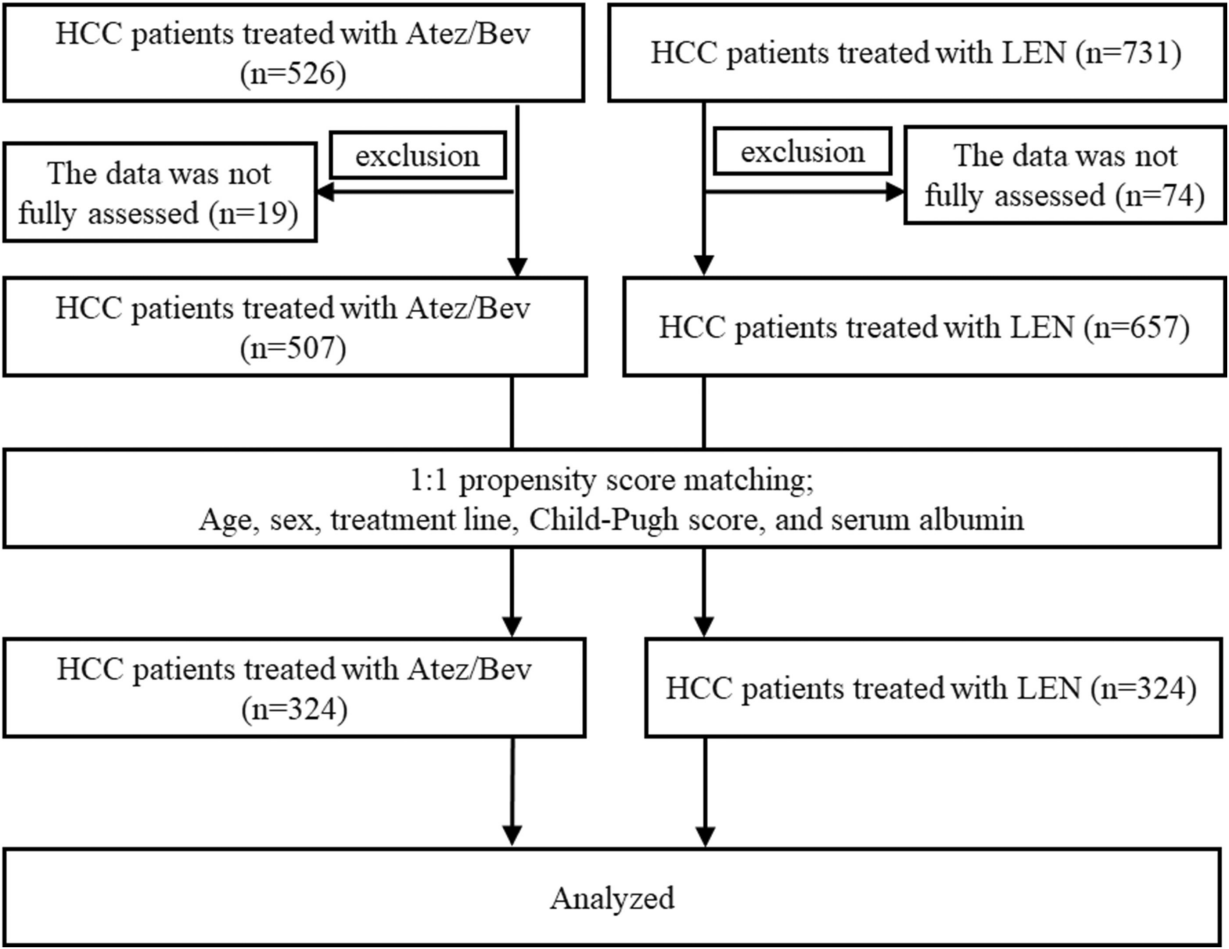
Patient selection process.

From March 2018 to July 2022, 526 patients with HCC received Atez/Bev and 731 patients received LEN at Japanese institutions. Among them, 19 patients in the Atez/Bev group, and 74 patients in the LEN group were excluded due to incomplete data. Among the remaining 507 patients in the Atez/Bev group and 657 patients in the LEN group, we conducted a 1:1 propensity‐score‐matched analysis to correct for patient imbalance between the two groups, resulting in the identification of 324 patients in each group for inclusion in the present analysis. A flow chart depicting the selection of patients is presented in Figure 1.

### 2.2 Atez/Bev and LEN treatments

The patients were administered atezolizumab at a dose of 1200 mg intravenously, followed by bevacizumab at a dose of 15 mg/kg once every 3 weeks. They typically visited the outpatient clinic every 3 weeks, and laboratory tests, including liver function assessment, were conducted before starting Atez/Bev treatment. The treatment‐related AEs were assessed based on the Common Terminology Criteria for Adverse Events version 5.0. In cases of unacceptable grade 2 or 3 AEs, we assessed the causal relationship between each drug (Atez or Bev) and the AEs, and temporarily interrupted the relevant drug. Generally, a reduction in the dose of atezolizumab and bevacizumab was not performed.

LEN was orally prescribed at a dose of 12 mg/day for patients with body weight (BW) ≥60 kg and 8 mg/day for patients with BW <60 kg. When the liver function is relatively poor, the initial dose of LEN may be reduced at the discretion of the attending physician. Patients generally visited the outpatient clinic every 4 weeks, and laboratory tests were conducted. If unacceptable grade 2 or 3 AEs were observed, we carried out dose reduction or temporary interruption of LEN.

In general, decisions regarding treatment, including the selection of systemic therapy agents, were determined based on clinical practice guidelines and the collective expertise of a multidisciplinary team at each hospital. This comprehensive approach aimed to ensure that the most appropriate and evidence‐based therapies were chosen for each patient's specific condition.

### 2.3 The evaluation of the liver function

Before starting each treatment, we evaluated the liver function using the Child–Pugh class. We also calculated the albumin‐bilirubin (ALBI) score with the following formula: (log_10_ bilirubin [μmol/L] × 0.66) + (albumin [g/L] × −0.085).^11^ Based on the calculated values, we also assessed the liver function using the modified ALBI grade (mALBI grade).^12^ During each treatment, we calculated the ALBI score to assess the changes of the liver function when the patients visited the outpatient clinic. We calculated the ALBI score at baseline, and at 3, 6, 9, and 12 weeks in the Atez/Bev group and at baseline, and at 4, 8, and 12 weeks in the LEN group.

### 2.4 Statistical analyses

Categorical variables were described as number (percentages) and were compared using the chi‐squared or Fisher's exact test, as appropriate. Continuous variables were reported as the medians [interquartile range] or mean ± standard error and compared using the Mann–Whitney *U*‐test. Because we found differences in patient characteristics between the two groups, we identified a propensity‐score‐matched cohort to minimize the risk of observational potential confounders. We created a propensity score for each patient by generating a logistic regression model using following variables; age, sex, treatment line (first and later line), Child–Pugh score, and serum albumin. We conducted 1:1 nearest neighbor matching without replacement and set a caliper width of 0.2 standard deviations.

We chose to use nonlinear mixed‐effects regression models because they can handle situations where treatment is discontinued within the first 12 weeks due to clinical reasons, including disease progression, serious AEs, or loss to follow‐up. It was treated as termination, when other treatment modalities, including transarterial chemoembolization and hepatic arterial infusion therapy, were introduced. To compare the changes in the ALBI score, and serum albumin and total bilirubin levels between the Atez/Bev and LEN groups, we used nonlinear mixed‐effects regression models to separately analyze each parameter. This allowed us to model the complex relationships between the variables of interest, account for individual differences and variation over time using random effects and compare the effects of the two treatments while controlling for potential confounding factors, even in cases where the timing of blood tests differed. All statistical analyses were conducted using EZR V. 1.55 (Saitama Medical Center, Jichi Medical University, Saitama, Japan).^13^

## 3 RESULTS

### 3.1 Patient characteristics before and after propensity score matching

The patient characteristics are listed in Table 1. The median ages of the Atez/Bev (*n* = 507) and LEN (*n* = 657) groups were 74.0 [68.0–80.0] and 73.0 [67.0–79.0] years old, respectively, with 408 (80.5%) and 529 (80.5%) being male, respectively. Approximately 80% of patients had a performance status of 0. About half of the patients had viral‐related liver diseases, and most patients were classified as BCLC intermediate or advanced stage. The percentage of patients with Child–Pugh class A was significantly higher in the Atez/Bev group than in the LEN group (*p* < 0.001). The serum albumin level was better in the Atez/Bev group than in the LEN group (*p* = 0.03). The proportion of patients receiving first‐line treatment was lower in the Atez/Bev group than in the LEN group. Regarding the initial dose, all patients in the Atez/Bev group received a full dose because dose reduction is not allowed in Atez/Bev treatment. In contrast, 198 patients (30.1%) in the LEN group initially received a reduced dose. The characteristics of the propensity‐score‐matched cohort are summarized in Table 2. No significant differences between the two groups were observed.

**TABLE 1:** Patient characteristics before propensity score matching.

		Atez/Bev group (*n* = 507)	LEN group (*n* = 657)	*p*‐value
Age		74.0 [68.0, 80.0]	73.0 [67.0, 79.0]	0.2
Gender, *n* (%)	Male	408 (80.5)	529 (80.5)	1.0
Performance status, *n* (%)	0	414 (81.7)	511 (77.8)	0.3
	1	78 (15.4)	124 (18.9)	
	≥2	15 (3.0)	22 (3.3)	
Chronic liver diseases, *n* (%)	HCV^a^	177 (34.9)	258 (39.3)	0.5
	HBV	87 (17.2)	100 (15.2)	
	Alcohol	101 (19.9)	125 (19.0)	
	Others	142 (28.0)	174 (26.5)	
BCLC stage, *n* (%)	Very early	7 (1.4)	7 (1.1)	0.9
	Early	25 (4.9)	34 (5.2)	
	Intermediate	188 (37.1)	234 (35.6)	
	Advanced	271 (53.5)	356 (54.2)	
	Terminal	16 (3.2)	26 (4.0)	
Child–Pugh class, *n* (%)	A	470 (92.7)	553 (84.2)	<0.001
	B	36 (7.1)	101 (15.4)	
	C	1 (0.2)	3 (0.5)	
ALBI score		−2.40 [−2.73, −2.08]	−2.39 [−2.70, −2.01]	0.1
Serum albumin (g/dL)		3.7 [3.3, 4.1]	3.6 [3.3, 4.0]	0.03
Total bilirubin (mg/dL)		0.8 [0.6, 1.1]	0.8 [0.6, 1.1]	0.6
mALBI grade, *n* (%)	1	182 (35.9)	215 (32.7)	0.4
	2a	125 (24.7)	168 (25.6)	
	2b	195 (38.5)	260 (39.6)	
	3	5 (1.0)	14 (2.1)	
Treatment line, *n* (%)	First line	322 (63.5)	489 (74.4)	<0.001
	Later line	185 (36.5)	168 (25.6)	
Initial dose, *n* (%)	Full dose	NA	459 (69.9)	NA
	Reduced dose	NA	198 (30.1)	
AFP	≥100 ng/mL	200 (39.4)	274 (41.7)	0.5
DCP	≥100 mAU/mL	322 (63.8)	311 (66.0)	0.5

Abbreviations: AFP, α‐fetoprotein; ALBI score, albumin‐bilirubin score; Atez/Bev, atezolizumab plus bevacizumab; BCLC stage, Barcelona Clinical Liver Cancer stage; DCP, Des‐gamma‐carboxy prothrombin; HBV, hepatitis B virus; HCV, hepatitis C virus; LEN, lenvatinib; mALBI grade, modified albumin‐bilirubin grade; NA, not available.

^a^ There were 130 (74.5%) and 102 patients (39.5%) who achieved sustained virological response in the Atez/Bev and LEN group, respectively.

**TABLE 2:** Patient characteristics after propensity score matching.

		Atez/Bev group (*n* = 324)	LEN group (*n* = 324)	*p*‐value
Age		74.0 [70.0, 80.0]	74.0 [69.0, 79.0]	1.0
Gender, *n* (%)	Male	276 (85.2)	276 (85.2)	1.0
Performance status, *n* (%)	0	274 (84.6)	262 (80.9)	0.4
	1	41 (12.7)	54 (16.7)	
	≥2	9 (2.8)	8 (2.5)	
Chronic liver diseases, *n* (%)	HCV^a^	115 (35.5)	129 (39.8)	0.7
	HBV	49 (15.1)	45 (13.9)	
	Alcohol	65 (20.1)	60 (18.5)	
	Others	95 (29.3)	90 (27.8)	
BCLC stage, *n* (%)	Very early	5 (1.5)	4 (1.2)	0.9
	Early	18 (5.6)	20 (6.2)	
	Intermediate	122 (37.7)	115 (35.5)	
	Advanced	170 (52.5)	172 (53.1)	
	Terminal	9 (2.8)	13 (4.0)	
Child–Pugh class, *n* (%)	A	310 (95.7)	310 (95.7)	1.0
	B	14 (4.3)	14 (4.3)	
	C	0 (0.0)	0 (0.0)	
ALBI score		−2.42 [−2.70, −2.15]	−2.48 [−2.73, −2.15]	0.4
Serum albumin		3.8 [3.4, 4.1]	3.8 [3.4, 4.0]	0.9
Total bilirubin		0.8 [0.6, 1.0]	0.8 [0.5, 1.0]	0.1
mALBI grade, *n* (%)	1	117 (36.1)	121 (37.3)	0.7
	2a	89 (27.5)	96 (29.6)	
	2b	117 (36.1)	105 (32.4)	
	3	1 (0.3)	2 (0.6)	
Treatment line, *n* (%)	First line	241 (74.4)	241 (74.4)	1.0
	Later line	83 (25.6)	83 (25.6)	
Initial dose, *n* (%)	Full dose	NA	236 (72.8)	NA
	Reduced dose	NA	88 (27.2)	
AFP	≥100 ng/mL	118 (36.4)	137 (42.3)	0.1
DCP	≥100 mAU/mL	200 (61.7)	166 (66.1)	0.3

Abbreviations: AFP, α‐fetoprotein; ALBI score, albumin‐bilirubin score; Atez/Bev, atezolizumab plus bevacizumab; BCLC stage, Barcelona Clinical Liver Cancer stage; DCP, Des‐gamma‐carboxy prothrombin; HBV, hepatitis B virus; HCV, hepatitis C virus; LEN, lenvatinib; mALBI grade, modified albumin‐bilirubin grade; NA, not available.

^a^ There were 83 (72.2%) and 54 patients (41.8%) who achieved sustained virological response in the Atez/Bev and LEN group, respectively.

### 3.2 The clinical outcomes and AEs of the Atez/Bev and LEN treatments

The objective response, as assessed by the modified Response Evaluation Criteria in Solid Tumors, presented in Table 3, and showed the following results: In the Atez/Bev group, there were 18 patients (5.6%) with a complete response, 104 patients (32.1%) with a partial response, 111 patients (34.3%) with stable disease, 63 patients (19.4%) with progressive disease, and 28 patients (8.6%) for whom response could not be evaluated. In the LEN group, the numbers were 20 patients (6.2%) with a complete response, 116 patients (35.8%) with a partial response, 112 patients (34.6%) with stable disease, 52 patients (16.0%) with progressive disease, and 24 patients (7.4%) for whom response could not be evaluated. The difference in response rates did not reach statistical significance (*p* = 0.7). The objective response rate was 37.7% in the Atez/Bev group and 42.0% in the LEN group, with no significant difference between the two groups (*p* = 0.3). Similarly, the disease control rate was 71.9% in the Atez/Bev group and 76.5% in the LEN group, and this difference also did not reach statistical significance (*p* = 0.2).

**TABLE 3:** Objective response in both groups.

	Atez/Bev group (*n* = 324)	LEN group (*n* = 324)	*p*‐value
Best response by mRECIST, *n* (%)			
CR	18 (5.6)	20 (6.2)	0.7
PR	104 (32.1)	116 (35.8)	
SD	111 (34.3)	112 (34.6)	
PD	63 (19.4)	52 (16.0)	
NE	28 (8.6)	24 (7.4)	
Objective response rate (%)	37.7	42.0	0.3
Disease control rate (%)	71.9	76.5	0.2

Abbreviations: Atez/Bev, atezolizumab plus bevacizumab; CR, complete response; LEN, lenvatinib; mRECIST, modified Response Evaluation Criteria in Solid Tumors; NE, not evaluated; PD, progressive disease; PR, partial response; SD, stable disease.

**FIGURE 2:**
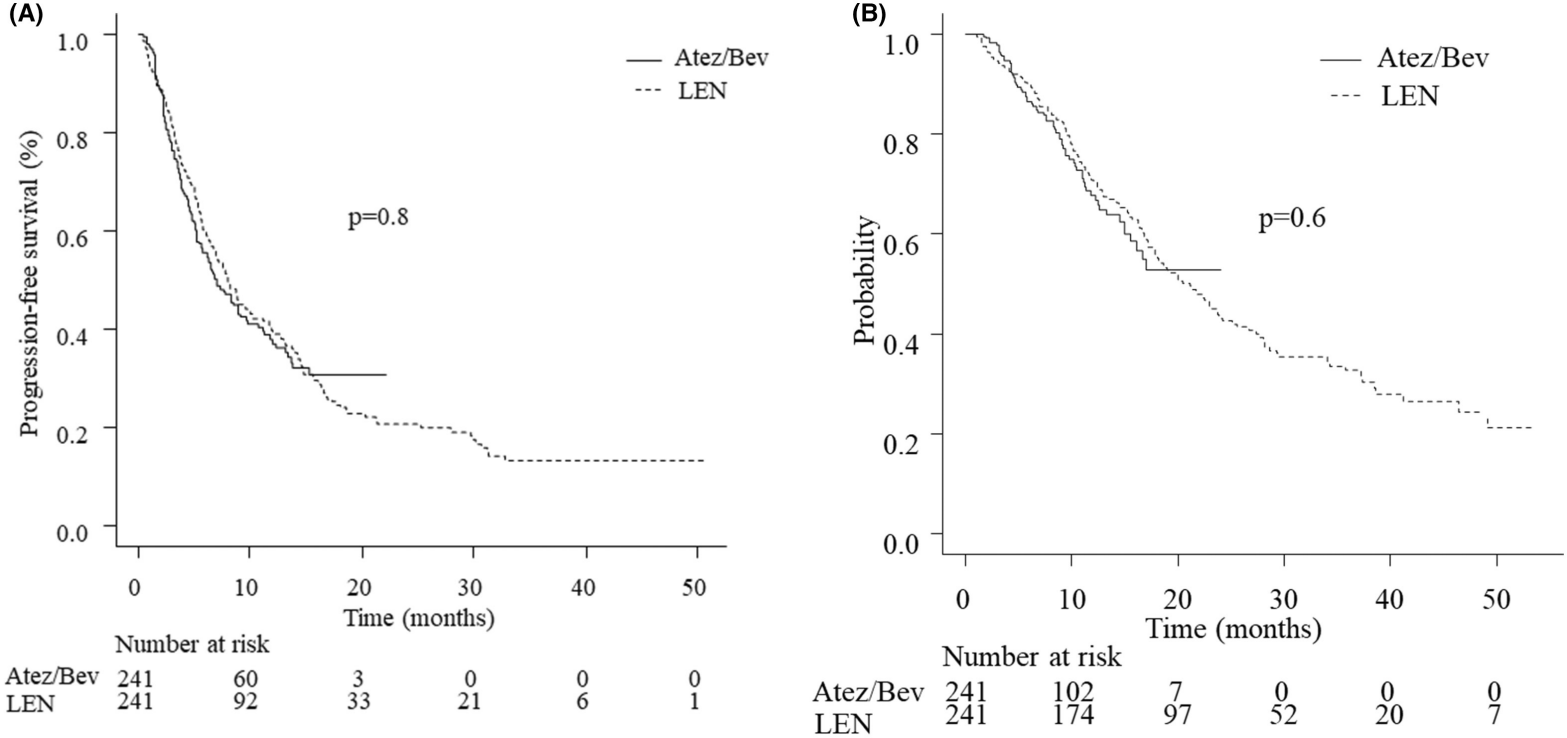
(a) Progression‐free survival. The median PFS in the patients who received the first‐line treatment was 7.0 months (95% CI 5.6–9.0) in the Atez/Bev group (*n* = 241) and 7.9 months (95% CI 6.7–9.7) in the LEN group (*n* = 241). No significant difference was observed between the two groups (*p* = 0.8). (b) Overall survival. The median OS was not reached in the Atez/Bev group, with a 1‐year survival rate of 67.7% (95% CI 59.8–74.3), while it was 21.1 months (95% CI 17.8–23.9) in the LEN group. The difference did not reach statistical significance (*p* = 0.6). Atez/Bev, atezolizumab and bevacizumab; CI, confidence interval; LEN, lenvatinib; OS, overall survival; PFS, progression‐free survival.

The median PFS in the patients who received the first‐line treatment was 7.0 months (95% CI 5.6–9.0) in the Atez/Bev group (*n* = 241) and 7.9 months (95% CI 6.7–9.7) in the LEN group (*n* = 241). No significant difference was observed between the two groups (*p* = 0.8; Figure 2A). The median OS was not reached in the Atez/Bev group, with a 1‐year survival rate of 67.7% (95% CI 59.8–74.3), while it was 21.1 months (95% CI 17.8–23.9) in the LEN group. The difference did not reach statistical significance (*p* = 0.6; Figure 2B).

In the Atez/Bev group, the most frequent AEs observed during the treatment were proteinuria (*n* = 103, 31.8%), followed by edema (*n* = 86, 26.5%), fatigue (*n* = 82, 25.3%), and appetite loss (*n* = 68, 21.0%). In the LEN group, appetite loss was the most common AEs (*n* = 113, 34.9%), followed by fatigue (*n* = 110, 34.0%), hand‐foot skin reaction (*n* = 95, 29.3%), and proteinuria (*n* = 79, 24.4%). The summary of AEs was shown in Table 4.

**TABLE 4:** Any adverse events observed during treatment in both groups (>10%).

	*n* = 324
Atez/Bev group, *n* (%)	
Liver injury	43 (13.3)
Hypertension	58 (17.9)
Rash	37 (11.4)
Appetite loss	68 (21.0)
Protein uria	103 (31.8)
Fatigue	82 (25.3)
Edema	86 (26.5)
LEN group, *n* (%)	
Diarrhea	59 (18.2)
Hypertension	72 (22.2)
Hand‐foot skin reaction	95 (29.3)
Appetite loss	113 (34.9)
Protein uria	79 (24.4)
Fatigue	110 (34.0)

Abbreviations: Atez/Bev, atezolizumab and bevacizumab; LEN, lenvatinib.

### 3.3 Analyses of changes in ALBI score in the Atez/Bev and LEN groups

**FIGURE 3:**
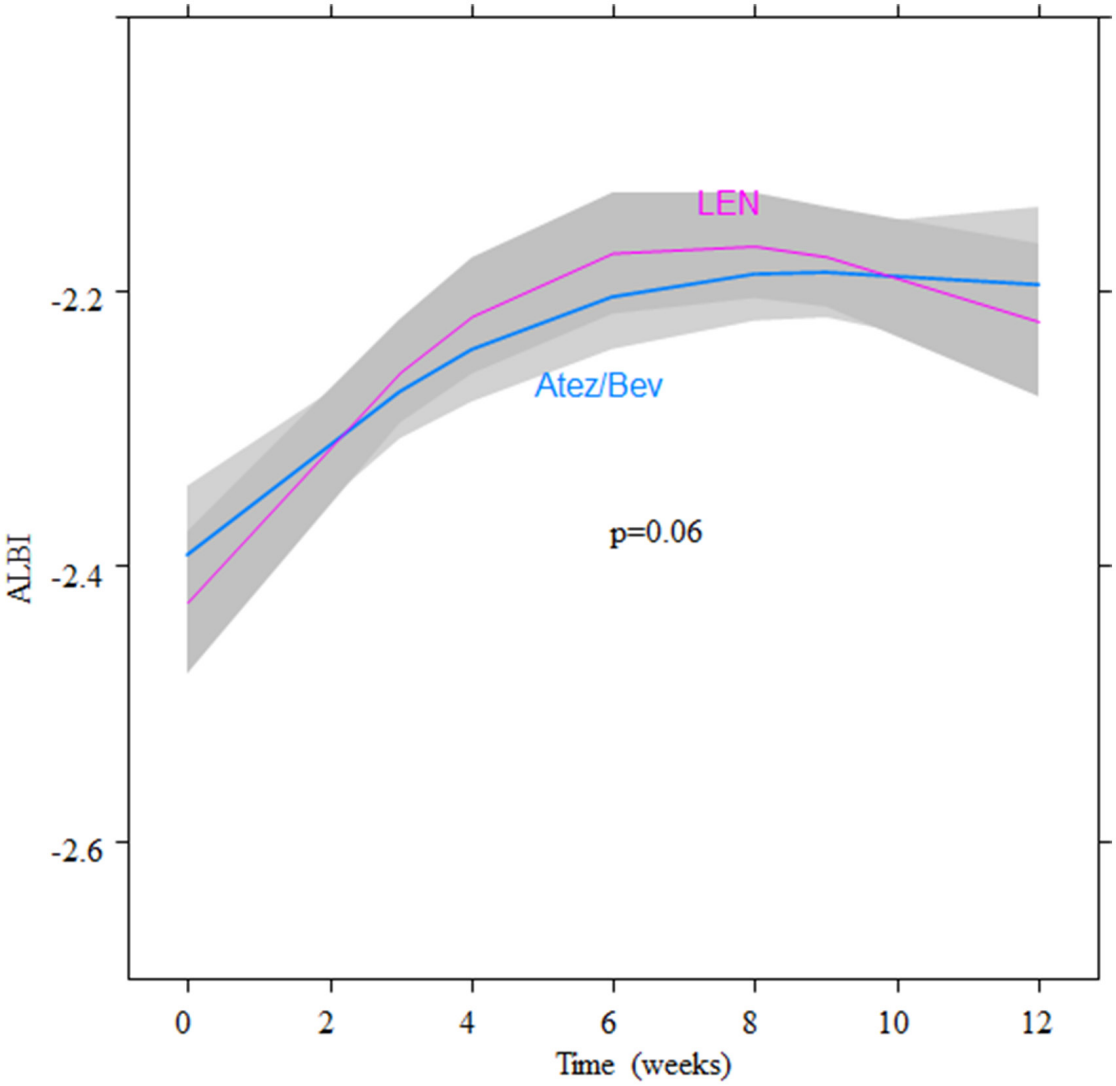
Time course of the albumin‐bilirubin score in the Atez/Bev and LEN groups by nonlinear mixed‐effects regression. The mean ALBI scores in the Atez/Bev and LEN groups were −2.41 ± 0.40 and −2.44 ± 0.42 at baseline, and −2.17 ± 0.56 and −2.19 ± 0.58 at 12 weeks, respectively. Although the ALBI score significantly worsened during treatment in both groups (*p* < 0.001), there was no significant difference in the rate of ALBI score deterioration between the groups (*p* = 0.06). The lines represent the mean of the obtained value and the shaded areas the 95% confidential interval. The blue and red lines indicate the Atez/Bev and LEN group, respectively. Atez/Bev, atezolizumab and bevacizumab; LEN, lenvatinib.

The changes in the ALBI score in both groups were shown in Figure 3. The mean ALBI score in the Atez/Bev and LEN groups was −2.41 ± 0.40 and −2.44 ± 0.42 at baseline, and −2.17 ± 0.56 and −2.19 ± 0.58 at 12 weeks, respectively. The ALBI score changed during the first roughly 8 weeks before reaching a plateau from approximately 8 to 12 weeks in both groups. Although the ALBI score significantly worsened during treatment in both groups (*p* < 0.001), there was no significant difference in the rate of ALBI score deterioration between the groups (*p* = 0.06).

### 3.4 Analyses of changes in the serum albumin and total bilirubin levels in the Atez/Bev and LEN groups

**FIGURE 4:**
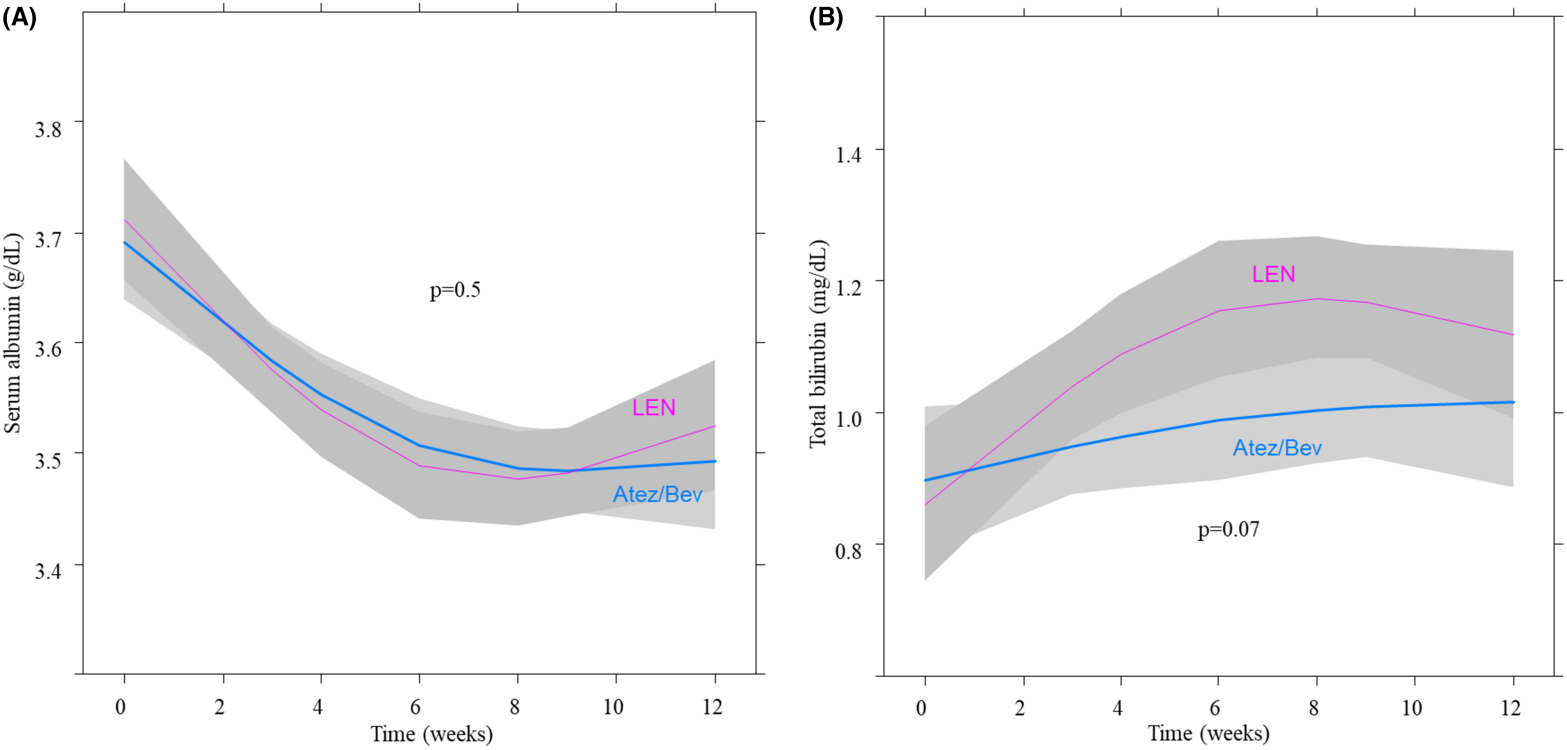
(a) Time course of the serum albumin level in the Atez/Bev and LEN groups by nonlinear mixed‐effects regression. At baseline, the mean serum albumin levels in the Atez/Bev and LEN groups were 3.7 ± 0.5 and 3.7 ± 0.5 g/dL, respectively. At 12 weeks, these levels decreased to 3.5 ± 0.6 and 3.5 ± 0.6 g/dL in the Atez/Bev and LEN groups, respectively. Although the serum albumin levels significantly decreased during treatment in both groups (*p* < 0.001), there was no significant difference in the rate of worsening serum albumin levels between the two groups (*p* = 0.5). (b) Time course of total bilirubin in the Atez/Bev and LEN groups by nonlinear mixed‐effects regression. At baseline, the mean total bilirubin levels in the Atez/Bev and LEN groups were 0.9 ± 0.4 and 0.8 ± 0.4 mg/dL, respectively. At 12 weeks, these levels increased to 1.0 ± 0.6 and 1.2 ± 2.0 mg/dL in the Atez/Bev and LEN groups, respectively. Although the total bilirubin levels significantly increased during treatment in both groups (*p* = 0.009), there was no significant difference in the rate of increase in total bilirubin levels between the groups (*p* = 0.07). The lines represent the mean of the obtained value and the shaded areas the 95% confidence interval. The blue and red lines indicate the Atez/Bev and LEN groups, respectively. Atez/Bev, atezolizumab and bevacizumab; LEN, lenvatinib.

The changes in the serum albumin and total bilirubin levels were described in Figure 4A,B. At baseline, the mean serum albumin levels in the Atez/Bev and LEN groups were 3.7 ± 0.5 and 3.7 ± 0.5 g/dL, respectively. At 12 weeks, these levels decreased to 3.5 ± 0.6 and 3.5 ± 0.6 g/dL in the Atez/Bev and LEN groups, respectively. Although the serum albumin levels significantly decreased during treatment in both groups (*p* < 0.001), there was no significant difference in the rate of decreasing serum albumin levels between the groups (*p* = 0.5).

At baseline, the mean total bilirubin levels in the Atez/Bev and LEN groups were 0.9 ± 0.4 and 0.8 ± 0.4 mg/dL, respectively. At 12 weeks, these levels increased to 1.0 ± 0.6 and 1.2 ± 2.0 mg/dL in the Atez/Bev and LEN groups, respectively. Although the total bilirubin levels significantly increased during treatment in both groups (*p* = 0.009), there was no significant difference in the rate of increase in total bilirubin levels between the groups (*p* = 0.07).

### 3.5 Subgroup analyses of changes in ALBI score in the Atez/Bev and LEN groups

**FIGURE 5:**
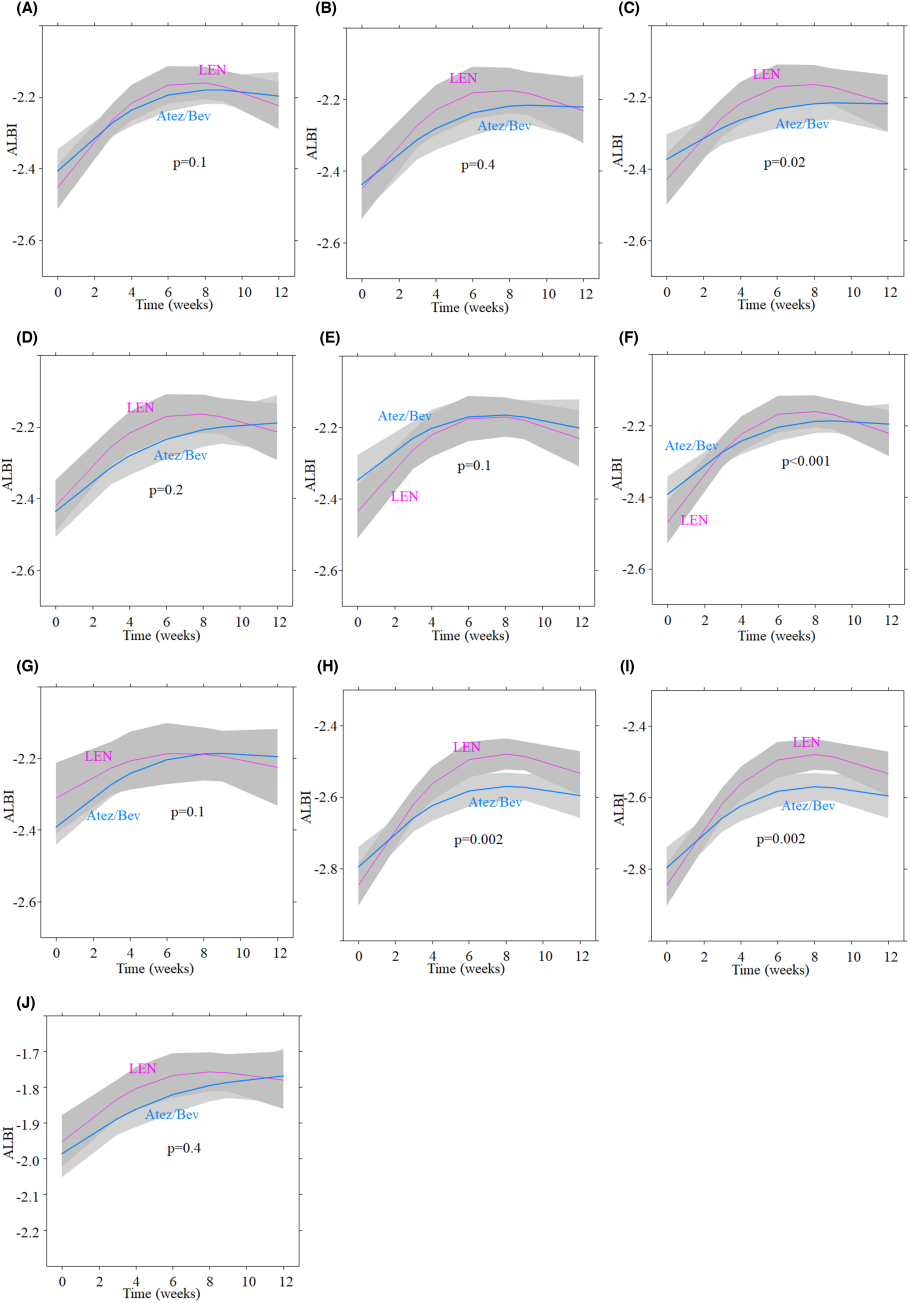
Subgroup analyses of changes in the albumin‐bilirubin score in the Atez/Bev and LEN groups. The ALBI score significantly worsened during treatment in both groups across all subgroups. Nonlinear mixed‐effects models showed no significant differences in patients receiving each treatment as first‐line treatment (*p* = 0.1, A) and those with BCLC intermediate stage (*p* = 0.4, B), but a significant difference was observed in patients with BCLC advanced stage (p = 0.02, C). There were no significant differences in the rate of ALBI score between the Atez/Bev and LEN groups in patients with viral and non‐viral infections (p = 0.2, D; *p* = 0.1, E). We compared the rate of ALBI score in patients who received Atez/Bev and those initially starting on the full dose of LEN, which resulted in a significant difference (*p* < 0.001, F). However, a significant difference was not observed in patients who received Atez/Bev and those initially starting on a reduced dose of LEN (*p* = 0.1, G). The ALBI score was significantly maintained in the Atez/Bev group compared to the LEN group in patients with mALBI grade 1 (*p* = 0.002, H) and those with mALBI grade 2a (*p* = 0.002, I), but not in those with mALBI grade 2b (*p* = 0.4, J). The lines represent the mean of the obtained value and the shaded areas the 95% confidence interval. The blue and red lines indicate the Atez/Bev and LEN groups, respectively. ALBI, albumin‐bilirubin; Atez/Bev, atezolizumab and bevacizumab; BCLC, Barcelona Clinical Liver Cancer stage; LEN, lenvatinib.

Next, we conducted subgroup analyses to assess changes in the ALBI score in the Atez/Bev and LEN groups. The time course of each subgroup analysis was presented in Figure 5A–J. The ALBI score significantly worsened during treatment in both groups across all subgroups. Nonlinear mixed‐effects models showed no significant differences in patients receiving each treatment as first‐line treatment (*p* = 0.1, Figure 5A) and those with BCLC intermediate stage (*p* = 0.4, Figure 5B), but a significant difference was observed in patients with BCLC advanced stage (*p* = 0.02, Figure 5C). There were no significant differences in the rate of ALBI score between the Atez/Bev and LEN groups in patients with viral and non‐viral infections, respectively (*p* = 0.2, Figure 5D; *p* = 0.1, Figure 5E). We compared the rate of ALBI score in patients who received Atez/Bev and those initially starting on the full dose of LEN (full dose group), which resulted in a significant difference (*p* < 0.001, Figure 5F). However, a significant difference was not observed in patients who received Atez/Bev and those initially starting on a reduced dose of LEN (*p* = 0.1, Figure 5G). The ALBI score showed significantly less deterioration in the Atez/Bev group than in the LEN group in patients with mALBI grade 1 (*p* = 0.002, Figure 5H) and those with mALBI grade 2a (*p* = 0.002, Figure 5I), but not in those with mALBI grade 2b (*p* = 0.4, Figure 5J).

Our study investigated the correlation between the initial dose of LEN and other factors, and found that the proportion of LEN‐treated patients receiving a full dose was significantly higher than that of those with a reduced dose in patients with mALBI grades 1 and 2a. However, the proportion of such patients receiving a full dose was significantly lower than that of those with a reduced dose in patients with mALBI grade 2b (*p* = 0.002, Table 5).

**TABLE 5:** Correlation between initial dose of LEN and treatment line, BCLC stage, chronic liver diseases, and mALBI grade.

		Initial dose of LEN		
		Full dose (*n* = 236)	Reduced dose (*n* = 88)	*p*‐value
Treatment line, *n* (%)	First line	173 (73.3)	68 (77.3)	0.6
BCLC stage, *n* (%)	Very early	4 (1.7)	0 (0.0)	0.2
	Early	11 (4.7)	9 (10.2)	
	Intermediate	80 (33.9)	35 (39.8)	
	Advanced	132 (55.9)	40 (45.5)	
	Terminal	9 (3.8)	4 (4.5)	
Chronic liver diseases, *n* (%)	Viral infection	102 (43.2)	48 (54.5)	0.08
mALBI grade, *n* (%)	1	97 (41.1)	24 (27.3)	0.002
	2a	75 (31.8)	21 (23.9)	
	2b	63 (26.7)	42 (47.7)	
	3	1 (0.4)	1 (1.1)	

Abbreviations: BCLC stage, Barcelona Clinical Liver Cancer stage; mALBI grade, modified albumin‐bilirubin grade.

## 4 DISCUSSION

Many previous studies on liver function have not considered cases of treatment interruption due to disease progression, adverse events, or loss to follow‐up. Excluding these cases could result in significant statistical biases. To address this issue, we employed a nonlinear mixed‐effects regression model, which allows for the evaluation and inclusion of these situations. To the best of our knowledge, this study is the first to analyze liver function, including cases of treatment interruption, using a nonlinear mixed‐effects regression model.

The main finding of this study is that nonlinear mixed‐effects models showed no statistically significant differences in the rate of ALBI score deterioration between the groups (*p* = 0.06), although the ALBI score significantly worsened during treatment in both groups (*p* < 0.001). The ALBI score changed during the first roughly 8 weeks before reaching a plateau from approximately 8–12 weeks in both groups. We also compared the rate of serum albumin and total bilirubin levels and demonstrated the lack of a significant difference in the rate of decrease in the serum albumin levels and increase in the total bilirubin levels between the groups (*p* = 0.5 and 0.07, respectively). Subgroup analyses showed significant differences in the rate of ALBI score among patients with BCLC advanced stage (*p* = 0.02), the full dose group (*p* < 0.001), and those with mALBI grade 1 (*p* = 0.002) and 2a (*p* = 0.04).

Most advanced HCC patients die from tumor progression or liver failure. To exclude the influence of liver failure, many randomized controlled trials exclude patients with Child–Pugh class B or C and include only patients with Child–Pugh class A. With the increasing availability of effective systemic therapies, it is important to evaluate the effect of these agents on the liver function. However, few studies have compared the effect of different agents on the liver function. Although Child–Pugh class is a well‐known method of evaluating the liver function, it consists of five variables, including serum albumin, total bilirubin and prothrombin time with cutoff values, and subjective assessments such as the presence of hepatic ascites and encephalopathy. In addition, frequent radiological imaging such as computed tomography and ultrasonography is required to evaluate changes in the hepatic ascites score. In contrast, the ALBI score consists of only 2 subjective variables and is a continuous variable without a cutoff value. Furthermore, the ALBI score is easy to evaluate changes in the liver function though blood test, without the need for radiological imaging. For these reasons, we chose the ALBI score to evaluate the time‐course changes in the liver function in the present study.

Following its introduction, Atez/Bev has been used as a first‐line treatment and LEN as a second‐line treatment following progression or discontinuation on Atez/Bev according to the HERITAGE study,^14^ which is a real‐world large population study in Japan. Accordingly, we chose the Atez/Bev and LEN among the seven available regimens and compared their effects on the liver function using nonlinear mixed‐effects models. We did not use a *t*‐paired test or ANOVA test because these statistical methods can only be used for complete cases and require exclusion of patients with missing data due to disease progression, treatment discontinuation, or loss to follow‐up. In addition, these statistical methods cannot be used for cases where the timing of blood tests differed. In fact, progressive disease at 6 weeks was observed in approximately 20% of patients receiving Atez/Bev, according to phase 1b study.^15^ To include these patients, we used nonlinear mixed‐effects models to assess the changes in the liver function.

The present study revealed that the ALBI score in the LEN group was significantly worsen among the patients with BCLC advanced stage, the full dose group, and those with mALBI grade 1 and 2a, compared to the Atez/Bev group. The reasons for the greater deterioration of liver function in the LEN group compared to the Atez/Bev group among these patients remain unclear. I and our colleagues previously reported that BCLC advanced stage was an unfavorable predictive factor associated with deterioration to Child–Pugh class B in patients receiving LEN.^16^ Accordingly, we speculated that the liver function might be more likely to worsen in BCLC advanced stage HCC patients treated with LEN than in those treated with Atez/Bev. LEN is an oral agent that acts as a multikinase inhibitor, including fibroblast growth factor (FGF) receptors 1–4.^17^, ^18^, ^19^ In a mouse model, FGF19 was associated with the regulation of hepatic protein and glycogen metabolism.^20^ Therefore, it is possible that high doses of LEN inhibit the production of albumin via FGFR inhibition, leading to an increase in the ALBI score in clinical settings. Indeed, the ALBI score and serum albumin showed significant deterioration in patients receiving a full dose of LEN compared to with those receiving a reduced dose (*p* < 0.001, and *p* < 0.001; Figure S1A,B). Because the proportion of LEN‐treated patients receiving a full dose was significantly higher than that of those with a reduced dose in patients with mALBI grades 1 and 2a, the ALBI score in the LEN group was significantly worsen among patients with mALBI grade 1 and 2a, compared to the Atez/Bev group.

Another thing to be noted was that there were not significant differences int the rate of ALBI score between the groups among patients with non‐viral infection. Pfister et al.^21^ reported that anti‐CD4‐anti‐PD1 treatment did not reduce the incidence of liver cancer and the non‐alcoholic fatty liver disease activity score in non‐alcoholic steatohepatits mouse model while anti‐CD8‐anti‐PD1 and anti‐TNF‐anti‐PD1 antibodies ameliorated liver damage, liver pathology, and liver inflammation. This report^21^ suggested that anti‐PD antibody exacerbated liver damage and liver inflammation and was likely to fail to maintain the liver function in NASH‐related HCC patients. However, based on our present results, anti‐PD antibody induced‐liver damage might be limited in clinical settings.

Several limitations associated with the present study warrant mention. This study was conducted in a retrospective manner with a relatively small sample size. Moreover, we evaluated the liver function only within the only first 12 weeks, and no evaluations of this function conducted subsequently. However, the liver function might not have changed beyond 12 weeks during both treatments, as our results showed that the ALBI score reached a plateau from 8 to 12 weeks in both groups. In this context, when both treatments were terminated due to disease progression, an increase in intrahepatic lesions might be attributed to the decline in liver function. Additionally, certain treatment‐related AEs, such as fatigue, loss of appetite, and proteinuria, have the potential to impede not only the continuation of both treatments but also subsequent therapies, ultimately resulting in an increase in intrahepatic lesions and a deterioration of liver function.

In conclusion, there was no significant difference in the liver function deterioration trend between Atez/Bev and LEN treatment. Caution might be required for LEN‐treated patients with BCLC advanced stage and those who initially received full dose.

## AUTHOR CONTRIBUTIONS

**Takeshi Hatanaka:** Conceptualization (lead); data curation (lead); formal analysis (lead); writing – original draft (lead). **Kakizaki Satoru:** Conceptualization (equal); data curation (equal); writing – review and editing (equal). **Atsushi Hiraoka:** Conceptualization (equal); data curation (equal); writing – review and editing (equal). **Toshifumi Tada:** Conceptualization (equal); data curation (equal); writing – review and editing (equal). **Masashi Hirooka:** Data curation (equal). **Kazuya Kariyama:** Data curation (equal); writing – review and editing (equal). **Joji Tani:** Data curation (equal). **Masanori Atsukawa:** Data curation (equal). **Koichi Takaguchi:** Data curation (equal). **Ei Itobayashi:** Data curation (equal). **Shinya Fukunishi:** Data curation (equal). **Kunihiko Tsuji:** Data curation (equal). **Toru Ishikawa:** Data curation (equal). **Kazuto Tajiri:** Data curation (equal). **Hironori Ochi:** Data curation (equal). **Satoshi Yasuda:** Data curation (equal). **Hidenori Toyoda:** Data curation (equal). **Chikara Ogawa:** Data curation (equal). **Keisuke Yokohama:** Data curation (equal). **Hiroki Nishikawa:** Data curation (equal). **Takashi Nishimura:** Data curation (equal). **Noritomo Shimada:** Data curation (equal). **Kazuhito Kawata:** Data curation (equal). **Hisashi Kosaka:** Data curation (equal). **Atsushi Naganuma:** Data curation (equal). **Yutaka Yata:** Data curation (equal). **Hideko Ohama:** Data curation (equal). **Hidekatsu Kuroda:** Data curation (equal). **Kazunari Tanaka:** Data curation (equal). **Takaaki Tanaka:** Data curation (equal). **Fujimasa Tada:** Data curation (equal). **Kazuhiro Nouso:** Data curation (equal). **Asahiro Morishita:** Data curation (equal). **Akemi Tsutsui:** Data curation (equal). **Takuya Nagano:** Data curation (equal). **Norio Itokawa:** Data curation (equal). **Tomomi Okubo:** Data curation (equal). **Taeang Arai:** Data curation (equal). **Michitaka Imai:** Data curation (equal). **Yohei Koizumi:** Data curation (equal). **Shinichiro Nakamura:** Data curation (equal). **Masaki Kaibori:** Data curation (equal). **Hiroko Iijima:** Data curation (equal). **Yoichi Hiasa:** Data curation (equal). **Masatoshi Kudo:** Writing – review and editing (equal). **Takashi Kumada:** Formal analysis (equal); writing – review and editing (equal).

## FUNDING INFORMATION

This study was not supported by external funding.

## CONFLICT OF INTEREST STATEMENT

Takeshi Hatanaka received lecture fees from Eisai. Atsushi Hiraoka received lecture fees from Eli Lilly, AstraZeneca, and Chugai. Toshifumi Tada received lecture fees from AbbVie, Eisai, and Chugai. Hidenori Toyoda received lecture fees from Eisai, Chugai, Takeda, Terumo, AbbVie, Gilead, Fujifilm WAKO, and Abott. Masatoshi Kudo received honoraria from Bayer, Chugai, Eisai, Eli Lilly, MSD, and Takeda; and received research funding from AbbVie, EA Pharma, Eisai, GE Healthcare, Gilead Sciences, Otsuka, Sumitomo Dainippon Pharma, Taiho, and Takeda. Takashi Kumada received lecture fees from Eisai. The other authors declare no conflicts of interest in association with the present study.

## ETHICS STATEMENT

Approval of the research protocol: This retrospective study was approved by the Institutional Ethics Committee of Japanese Red Cross Himeji Hospital (IRB No. 2022‐40) in accordance with the Declaration of Helsinki.

## Supporting information


Figure S1.

## Data Availability

All data supporting the present study will be available from the corresponding author upon reasonable request.
