# Supplementary figures and images for: Comparing the impact of atezolizumab plus bevacizumab and lenvatinib on the liver function in hepatocellular carcinoma patients: A mixed‐effects regression model approach
Source: Cancer Med. 2023 Nov 21;12(24):21680–93. doi: 10.1002/cam4.6726 (PMC10757119; doi:10.1002/cam4.6726)

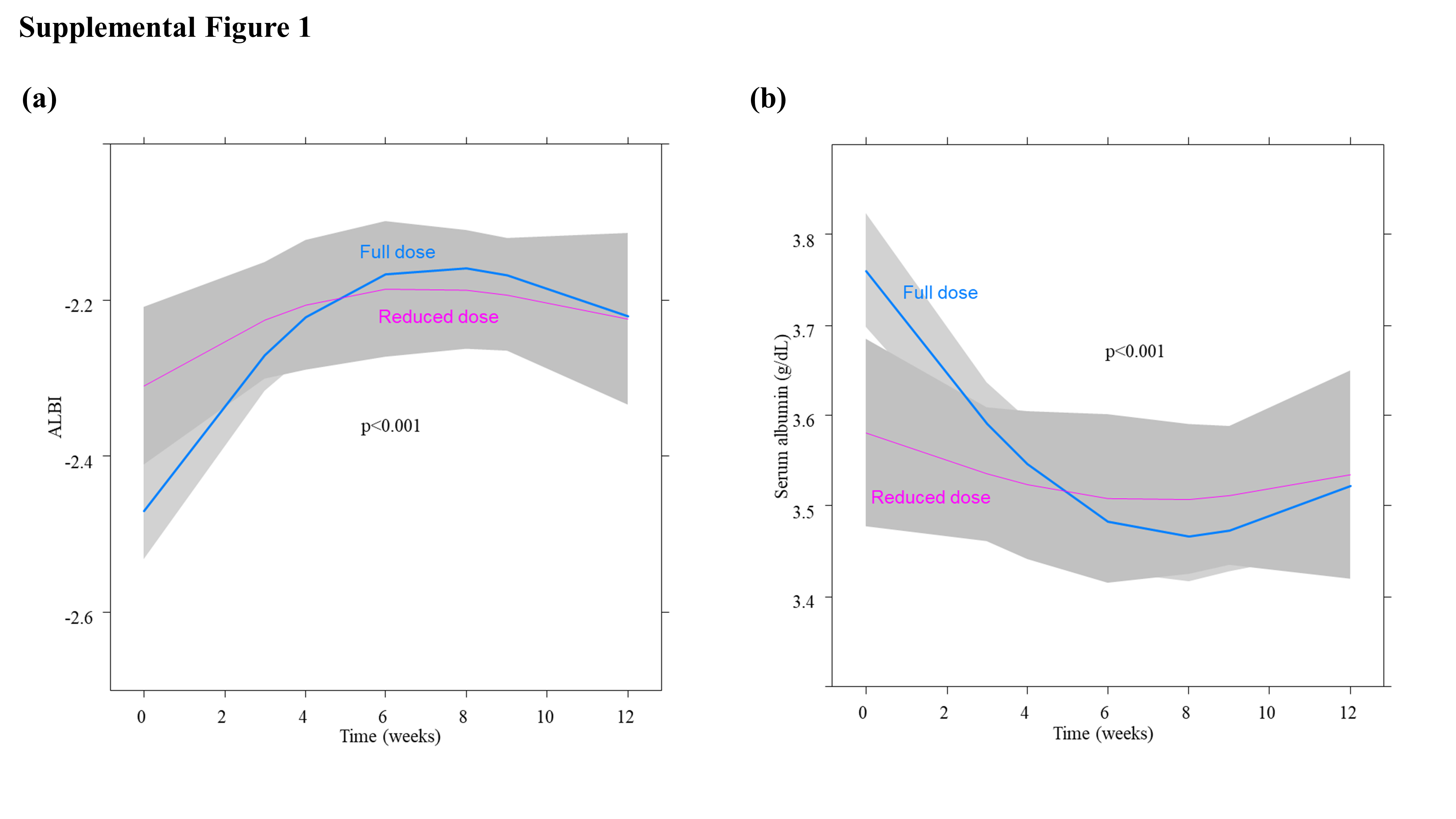

Supplement: Supplementary file 1 — Figure S1. [file CAM4-12-21680-s001.tif]
